# Supplementary material for: Automated sample preparation with SP3 for low‐input clinical proteomics
Source: Mol Syst Biol. 2020 Jan 16;16(1):e9111. doi: 10.15252/msb.20199111 (PMC6966100; doi:10.15252/msb.20199111)
Supplement: Supplementary file 1 — Appendix [file MSB-16-e9111-s001.pdf]

Table of Content

Appendix Figure S1. Manual SP3 versus autoSP3. 2
Appendix Figure S2. Experimental validation of absence of cross-contamination. 3
Appendix Figure S3. Protein identifications during longitudinal sampling. 4
Appendix Figure S4. Comparison of coefficient of variation (CV) distribution between manually and automatically prepared samples. 5
Appendix Figure S5. Protein abundance and coefficient of variation (CV). 6
Appendix Figure S6. Pulmonary adenocarcinoma (ADC) FFPE tissue proteomics. 7
Appendix Figure S7. Differential expression analysis between growth patterns. 8
Appendix Figure S8. Gene ontology network analysis and GSEA. 10
Appendix Figure S9. Differential expression analysis of papillary subgroups. 10

Appendix Protocol A. Single-step reduction and alkylation with TCEP/ CAA plus core autoSP3 clean-up. 11
Appendix Protocol B. Two-step reduction and alkylation with e.g. DTT/ CAA plus core autoSP3 clean-up. 14
Appendix Protocol C. Core autoSP3 clean-up (omitting automated reduction and alkylation). 15
Appendix Protocol D. Acidification and recovery of peptides to a new sample plate. 16

The instrument files (*.vzp files) for the methods on the Agilent Bravo system corresponding to Protocols A to D have been deposited to the ProteomeXchange Consortium via the PRIDE partner repository with the dataset identifier PXD014556.

Appendix Figures

Appendix Figure S1

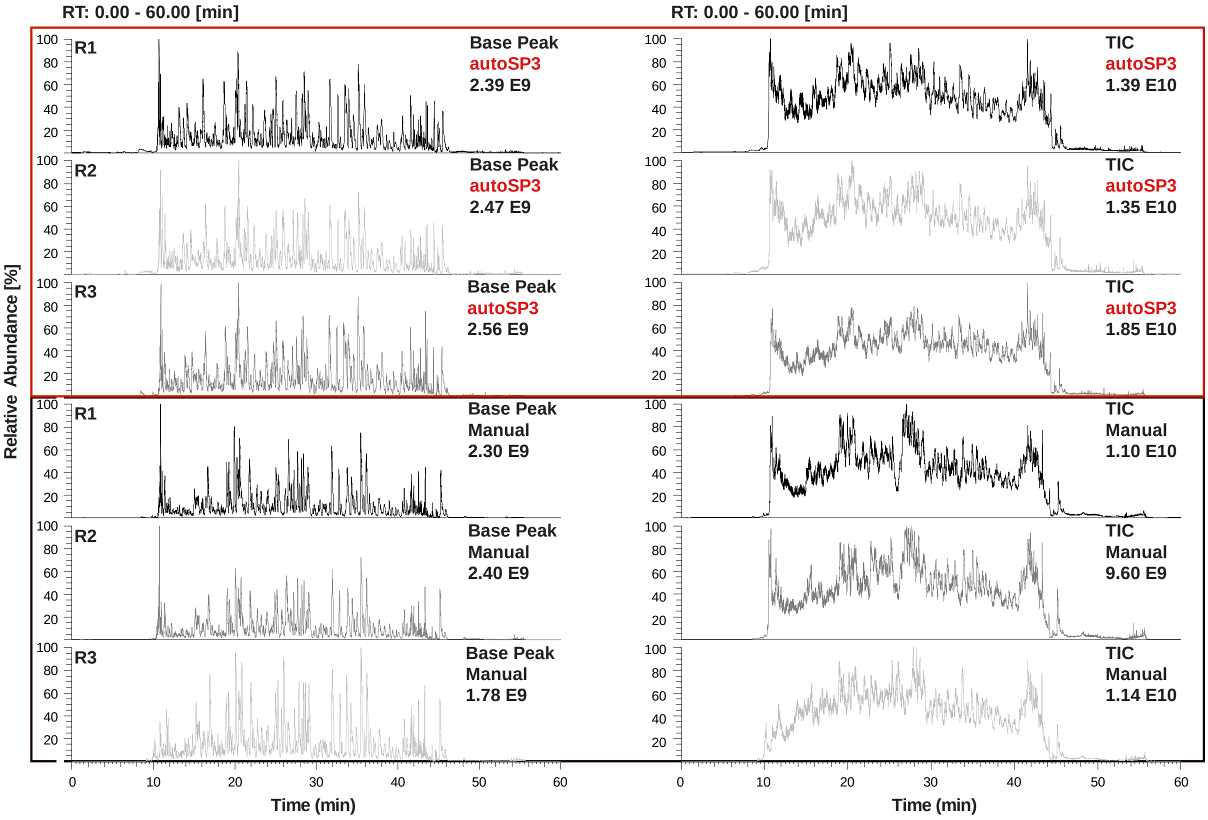

Method	Replicates	Peptide Sequences Identified	LFQ proteins
autoSP3	HeLa R1	12163	2730
	HeLa R2	11709	2733
	HeLa R3	12582	2779
Manual SP3	HeLa R1	11207	2665
	HeLa R2	11849	2713
	HeLa R3	11774	2714

Appendix Figure S 1. Manual SP3 versus autoSP3.
A comparison of three versus three individually processed samples using the manual SP3 protocol or autoSP3. The upper panel (red) shows the base peak and total ion chromatogram (TIC) of three autoSP3 HeLa samples, while the lower panel (black) shows base peak and TIC of three manual SP3 HeLa samples. The number of proteins and peptides identified by either workflow is indicated per replicate.

Appendix Figure S2

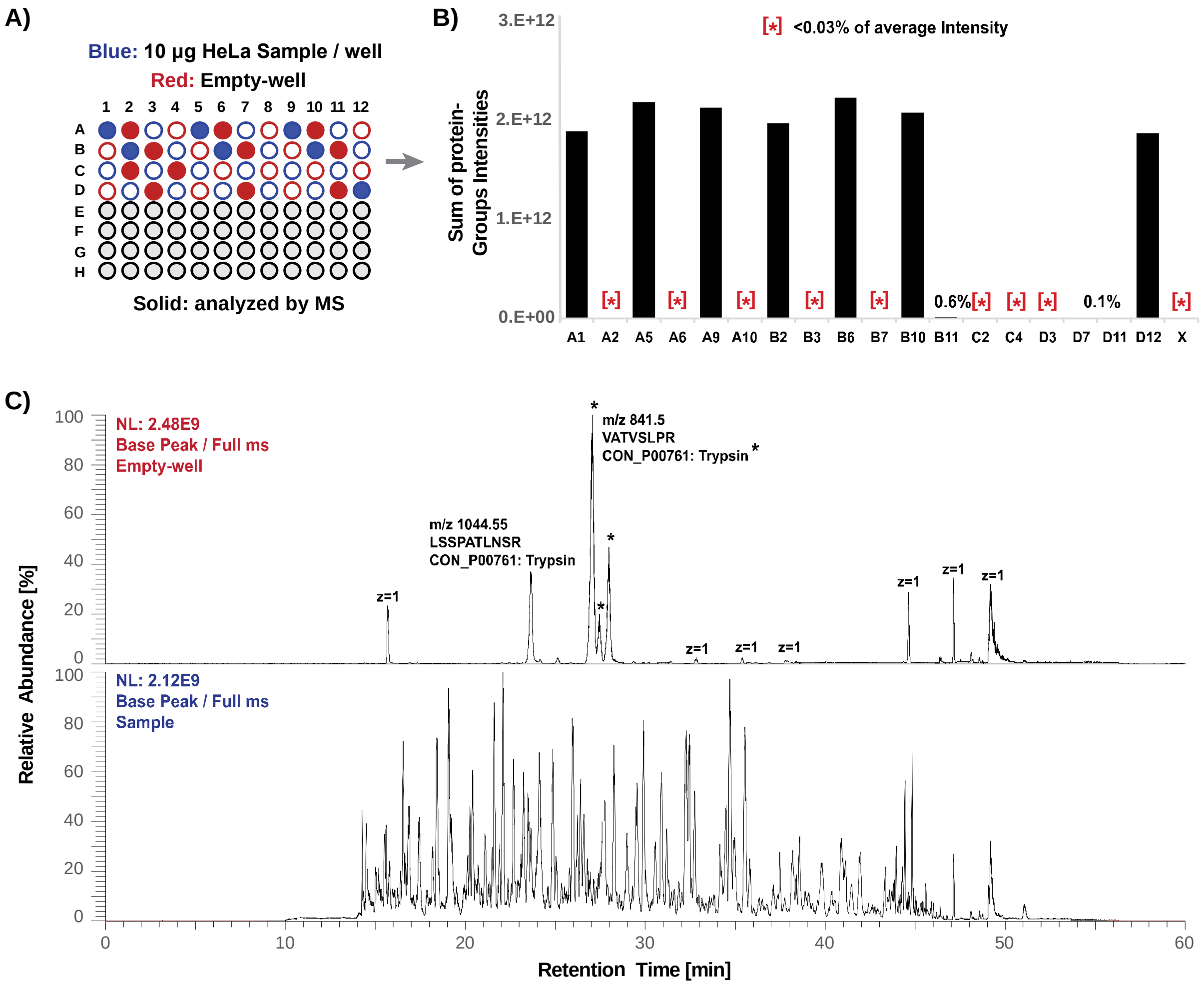

Appendix Figure S 2. Experimental validation of absence of cross-contamination.
A) Schematic representation of the experimental design to demonstrate the absence of cross contamination between wells. Half a plate (48 wells) was processed with 10 µg protein of a HeLa batch lysate in every second well (highlighted in blue) interspaced with empty wells as a control (highlighted in red). Randomly selected wells (highlighted in solid) were selected for direct LCMS. B) Bar plots of the summed intensities of protein groups across selected samples. A total of seven sample-containing injections were performed and a total of twelve empty controls. Asterisks indicate intensities <0.03%. C) Exemplary base peak MS1 spectrum for an empty control injection (top) and a sample-containing injection (bottom).

Appendix Figure S3

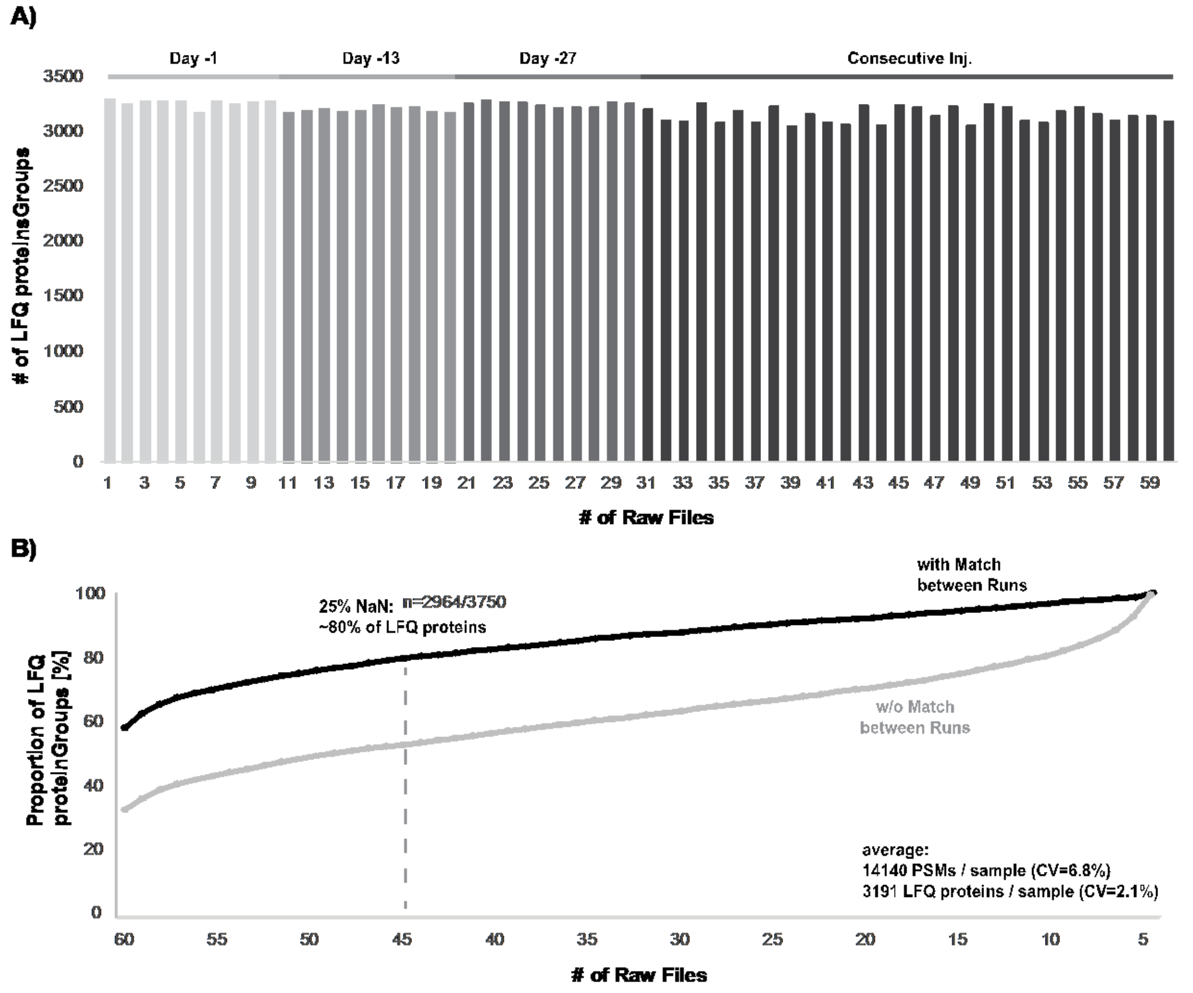

Appendix Figure S 3. Protein identifications during longitudinal sampling.
A) Bar plot summarizing the number of quantified LFQ protein groups across 60 HeLa samples. Samples originating from different days and the consecutive injections of the same samples are highlighted in grey scales. B) A line chart showing the proportion of quantified proteinGroups across all 60 autoSP3 HeLa samples. The data are shown with and without the use of match-between-runs.

Appendix Figure S4

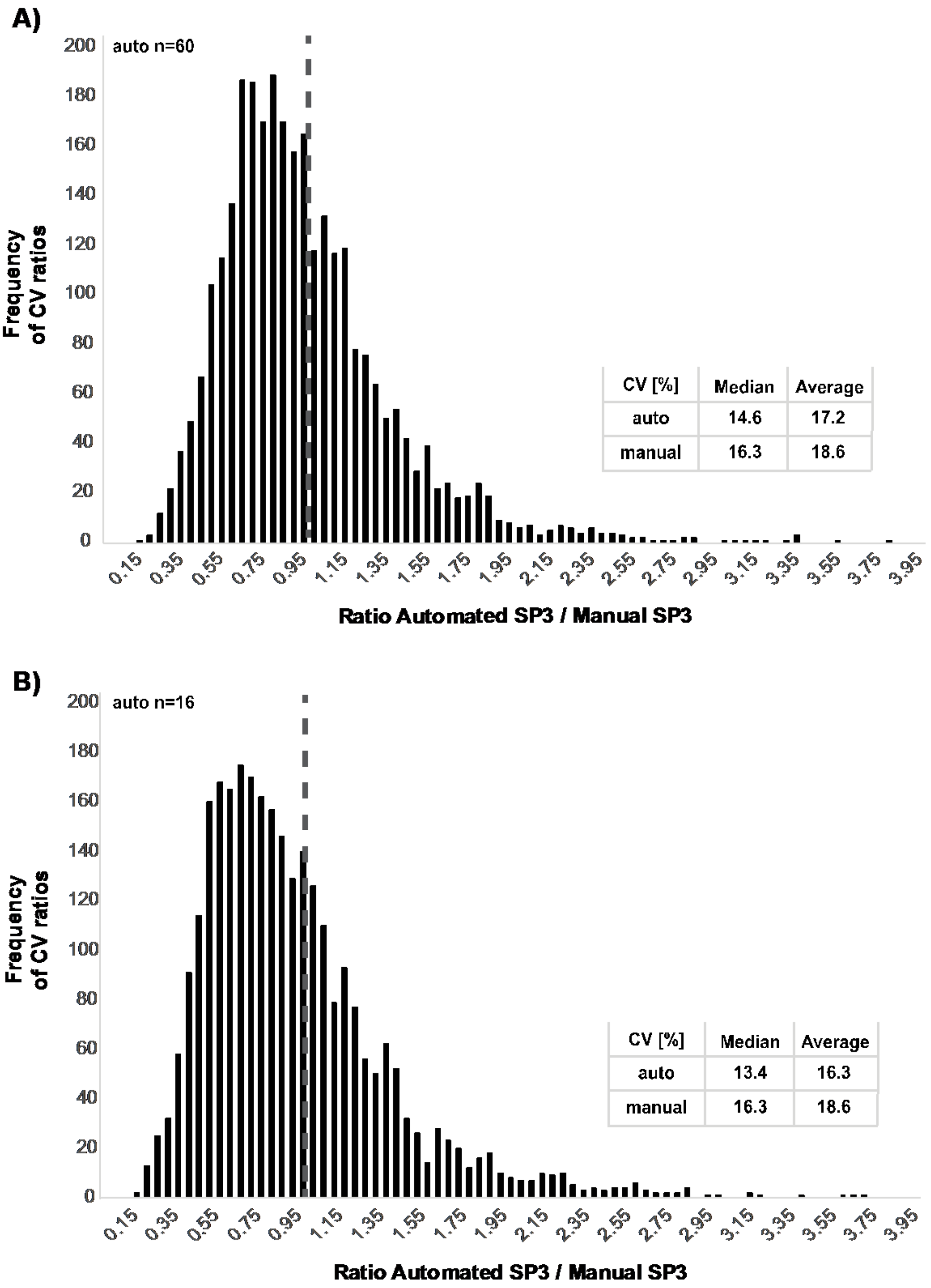

Appendix Figure S 4. Comparison of coefficient of variation (CV) distribution between manually and automatically prepared samples.
A) Histogram showing CVs of quantified proteins across all 60 automatically prepared HeLa samples, proportional to CVs of sixteen manually prepared samples. The median and average CV is shown for both automatically and manually prepared samples. A dotted line highlights the ratio of 1. B) Histogram showing CVs of quantified proteins from sixteen randomly selected out of 60 samples, proportional to

sixteen manually prepared samples. The median and average CV is shown for both, automatically and manually prepared samples. A dotted line highlights the ratio of 1.

Appendix Figure S5

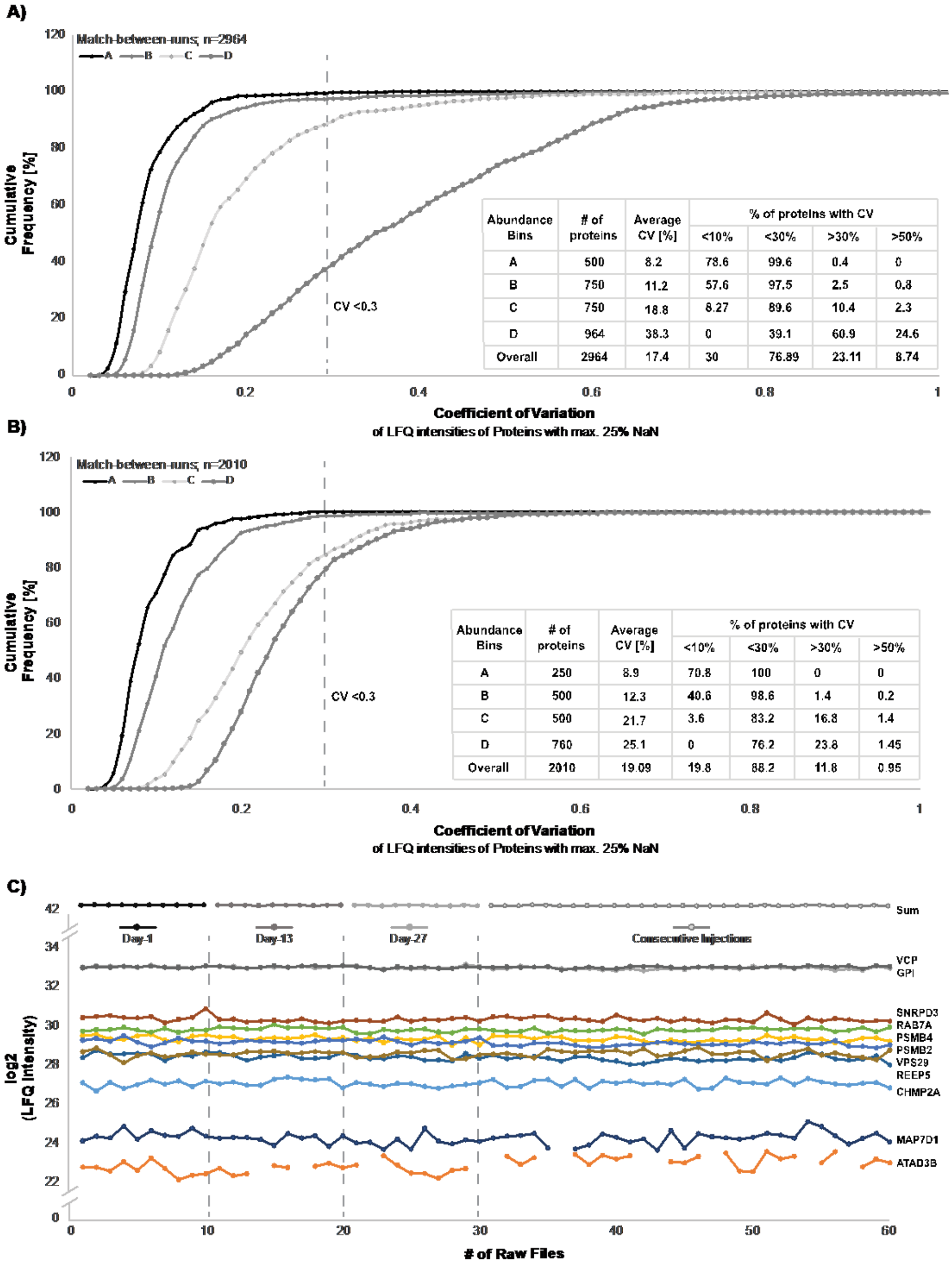

Abundance Bins	# of proteins	Average CV [%]	% of proteins with CV			
			<10%	<30%	>30%	>50%
A	500	8.2	78.6	99.6	0.4	0
B	750	11.2	57.6	97.5	2.5	0.8
C	750	18.8	8.27	89.6	10.4	2.3
D	964	38.3	0	39.1	60.9	24.6
Overall	2964	17.4	30	76.89	23.11	8.74

Abundance Bins	# of proteins	Average CV [%]	% of proteins with CV			
			<10%	<30%	>30%	>50%
A	250	8.9	70.8	100	0	0
B	500	12.3	40.6	98.6	1.4	0.2
C	500	21.7	3.6	83.2	16.8	1.4
D	760	25.1	0	76.2	23.8	1.45
Overall	2010	19.09	19.8	88.2	11.8	0.95

Appendix Figure S 5. Protein abundance and coefficient of variation (CV).

A) Four protein abundance bins (A, B, C, and D) were defined and cumulative frequency distributions [%] of the calculated CVs of quantified proteins (including match-between-runs) within each bin are plotted. The corresponding average CV values per group are shown. The table summarizes the percentage of quantified proteins observed with a CV higher or lower than 10%, 30%, and 50% for each abundance bin. B) Same as in A, the data are plotted without the use of match-between-runs. C) log_2 LFQ intensities of selected individual proteins and the sum of all proteins within a sample are plotted across all 60 measurements.

Appendix Figure S6

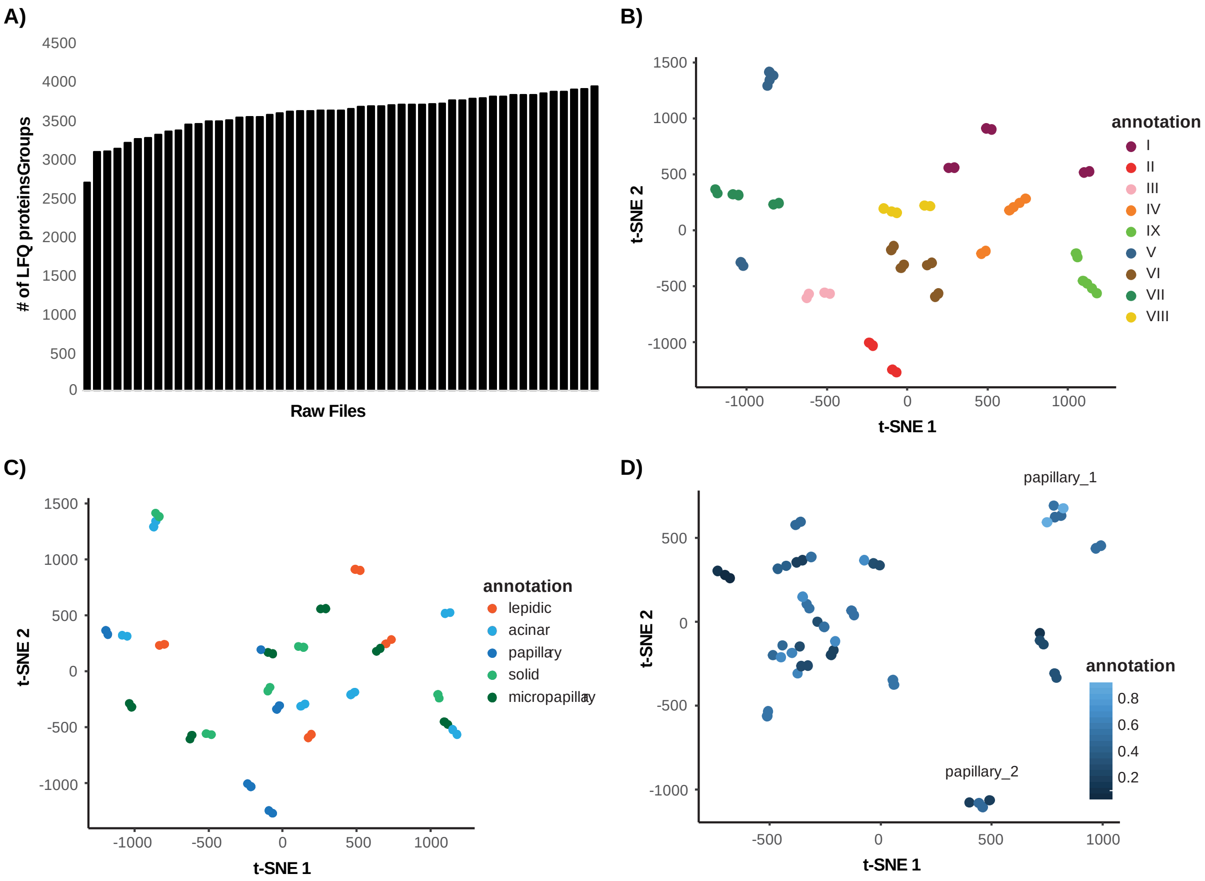

Appendix Figure S 6. Pulmonary adenocarcinoma (ADC) FFPE tissue proteomics.
A) Bar plot summarizing the number of quantified LFQ protein groups per sample. B) t-distributed stochastic neighbor embedding (t-SNE) analysis of the uncorrected proteome data. The samples are color-coded according to their patient origin. C) Same as in B, now color-coded according to their tumor growth pattern. D) Same as in Figure 4B, now color-coded for the tumor cell content (TCC) [%] of each sample.

Appendix Figure S7

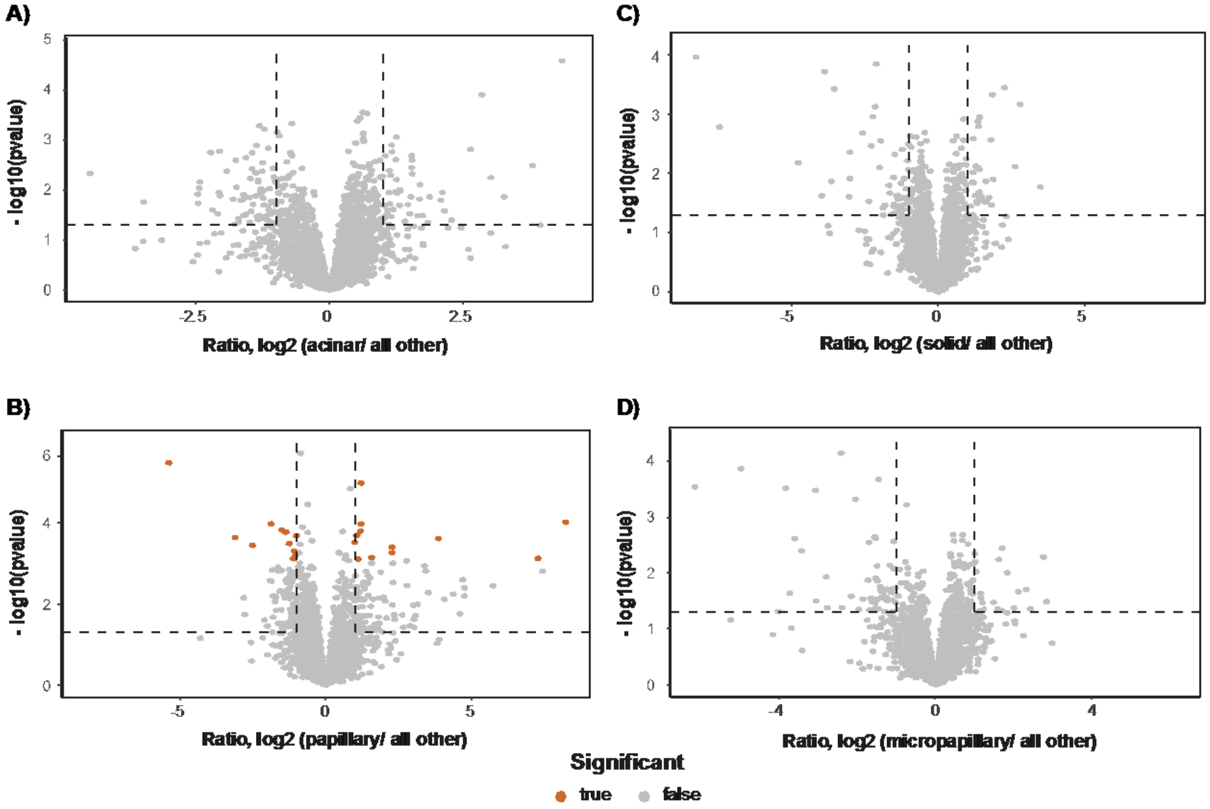

Appendix Figure S 7. Differential expression analysis between growth patterns.
Corresponding to Figure 5, showing the differential expression analysis of acinar (A), papillary (B), solid (C), and micropapillary (D) tumors compared to all other samples by using Limma moderated t-statistics. Proteins passing significance thresholds of $-log_{10}$ p-value < 0.05 (Benjamini-hochberg adjusted) and an absolute log_2 fold change of 1 are highlighted in orange.

Appendix Figure S8

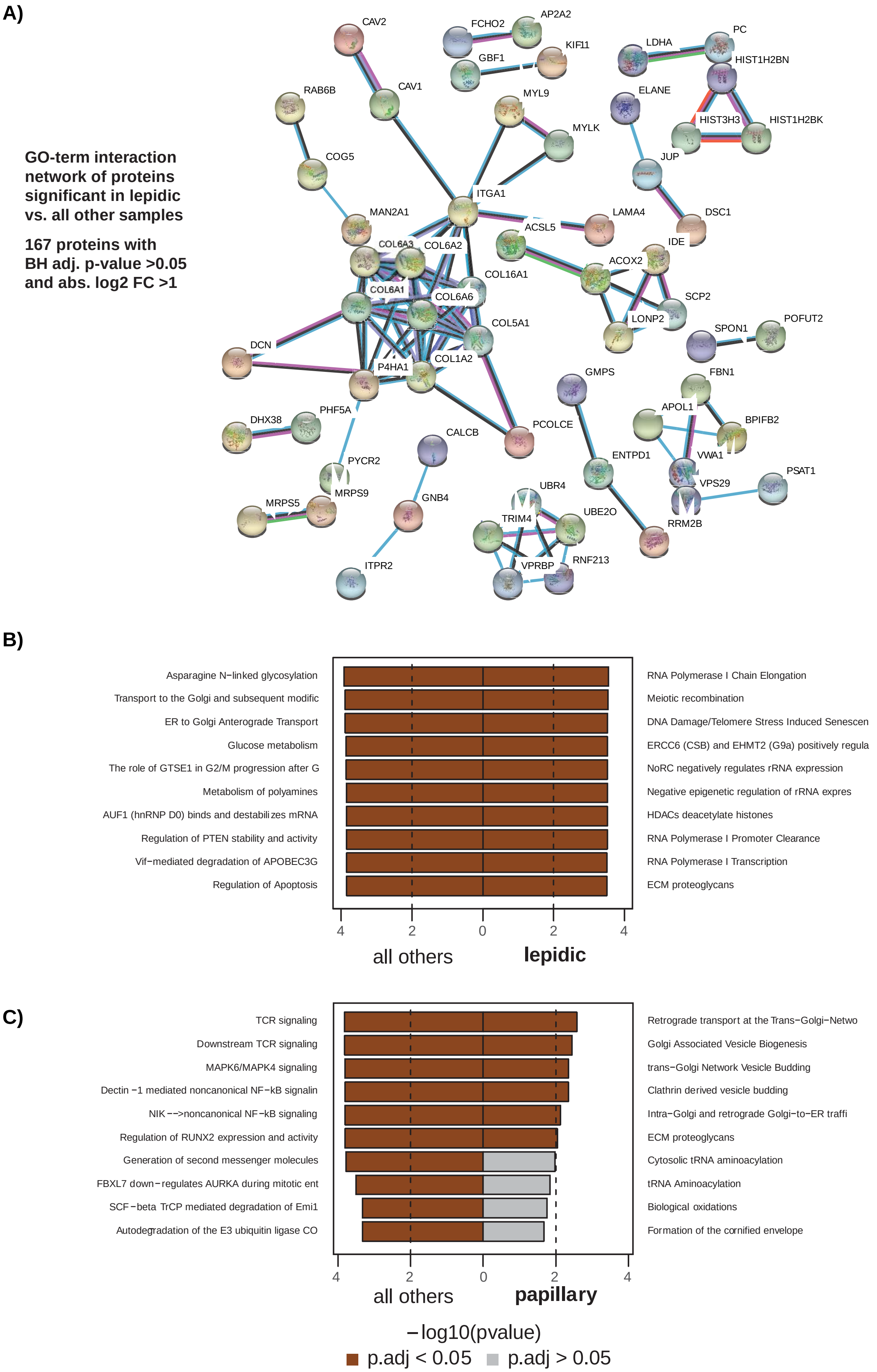

Appendix Figure S 8. Gene ontology network analysis and GSEA.
A) STRING network analysis of the 167 significant proteins (-log_{10} p-value < 0.05 and an absolute log_2 fold change >1) in lepidic versus all other samples. B) Gene set enrichment analysis of p-value ranked proteins for lepidic versus all other samples. C) Gene set enrichment analysis of p-value ranked proteins for papillary versus all other samples. In both GSEA analyses, gene sets with an adjusted -log_{10} p-value < 0.05 were considered significant and are highlighted in dark color.

Appendix Figure S9

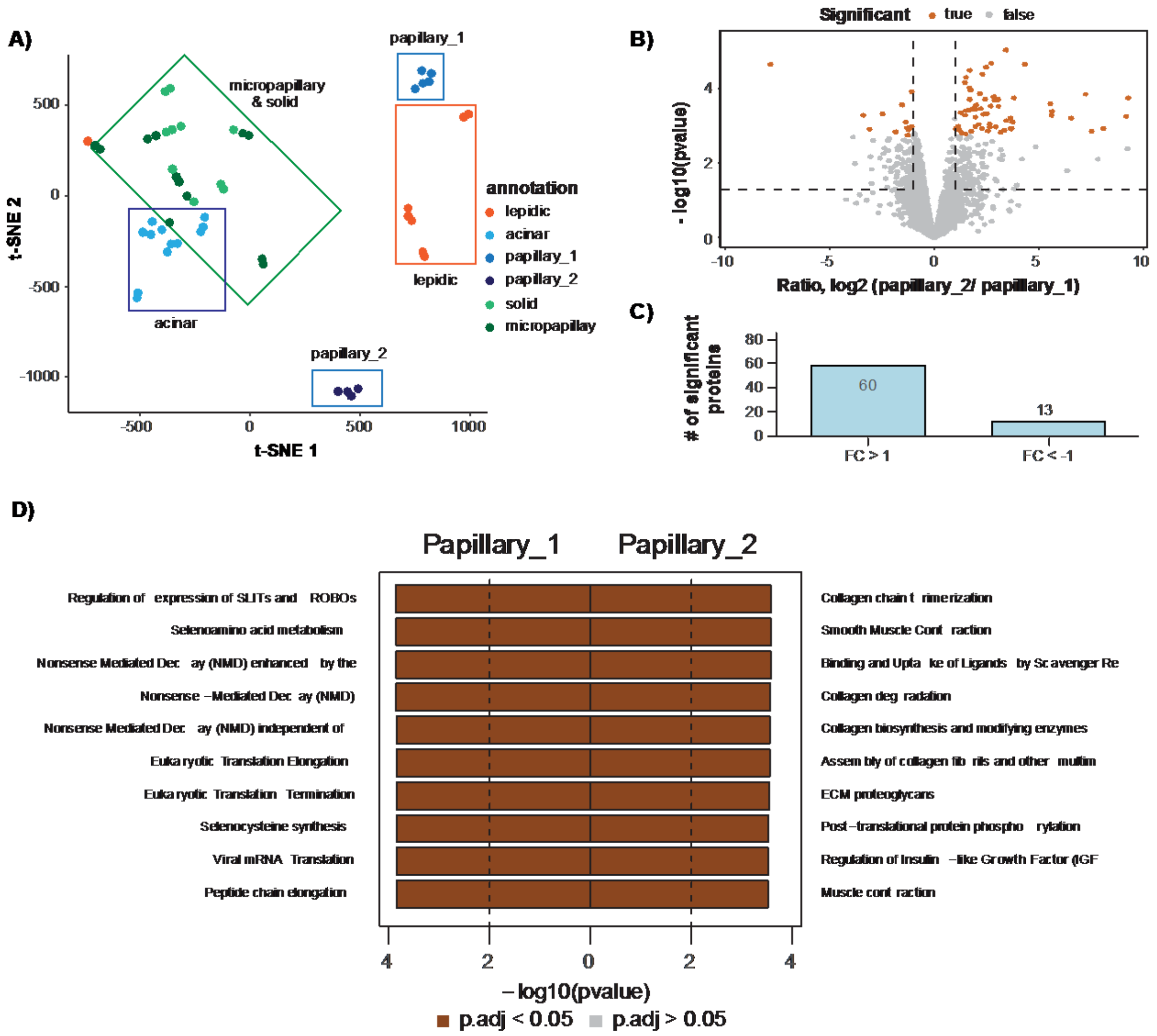

Appendix Figure S 9. Differential expression analysis of papillary subgroups.
A) t-distributed stochastic neighbor embedding (t-SNE) analysis of the proteome data corrected using a linear regression model. B) Differential expression analysis between subclusters papillary_1 and papillary_2 (see A) using Limma moderated t-statistics. Proteins passing significance thresholds of -log_{10} p-value < 0.05 05 (Benjamini-hochberg adjusted) and an absolute log_2 fold change >1 are highlighted in orange. C) The number of differentially expressed proteins in the papillary subcluster comparison. D) Gene set enrichment analysis of p-value ranked proteins for papillary_1 versus papillary_2. Gene sets with an adjusted -log_{10} p-value < 0.05 were considered significant and are highlighted in dark color.

Appendix Protocols

Appendix autoSP3 ProtocolA: Single-step Reduction andAlkylation with TCEP/ CAA + core SP3 clean-up

Protocol A	Liquid Handling	Plate Movement	Total Time
Reduction & Alkylation	1: Starting Heating of Position 6 to 95°C	1: -	2 sec
	2: TCEP/CAA reagent mix is transfered row-by-row to the Sample Plate (42 µL aspirated; 5 µL dispensed per well)	2: Bead/Trypsin Plate is moved to Position 5 to allow accessibility of Row C. Sample Plate is moved to Position 6 to allow dispensing across the entire plate. Subsequently Bead/Trypsin Plate is returned to Position 9	2 min 46 sec
	3: Hanging droplet of TCEP/CAA reagent is moved into the Sample Solution by shaking at 1500 rpm for 30 seconds	3: Sample Plate is moved to Orbital Shaking Station (Position 7)	3 min 27 sec
	4: Wait for 25 minutes to finish heating to of Position 6 to 95°C	4: Sample Plate is moved to Position 5	28 min 50 sec
	5: 5 minutes at 95°C for sufficient Reduction and Alkylation	5: Sample Plate is moved to Position 6	34 min
	6: Wait for ~12 minutes to cool of Position 6	6: Sample Plate is moved to Position 5	~ 46 min
Protein Binding	7: Magnetic Bead stock solution is transfered row-by-row to the Sample Plate (42 µL aspirated; 5 µL dispensed per well)	7: Sample Plate is moved to Position 6 to allow dispensing across entire plate	48 min 3 sec
	8: Hanging droplet of Magnetic Beads is moved into the Sample Solution by shaking at 1500 rpm for 20 seconds	8: Sample Plate is moved to Orbital Shaking Station (Position 7)	48 min 43 sec
	9: Acetonitrile (100%) is transfered from Position 2 to Sample Plate (137 µL aspirated; 15 µL dispensed per well)	9: Sample Plate is moved to Position 6 to allow dispensing across entire plate	52 min 53 sec
	10: Proteins are allowed to bind to the Magnetic Beads while shaking in nine consecutive iterations of 30 seconds at 1500 rpm & 90 seconds at 100 rpm	10: Sample Plate is moved to Orbital Shaking Station (Position 7)	1 hour 11 min 21 sec
	11: Bead-bound Proteins are trapped on the Magnetic Rack for 5 minutes to allow subsequent supernatant removal	11: Sample Plate is moved to Magnet (Position 5)	1 hour 16 min 31 sec
	12: Removal of supernatant in two consecutive pipetting steps using well-specific tips from tip box in Position 3 (1x 18 µL & 1x 25 µL). Dispensing in Waste Container (Position 8)	12: Supernatant is removed while remaining on Magnet (Position 5)	1 hour 18 min 56 sec
Ethanol wash	13.1: Addition of 200 µL 80% Ethanol (Position 2): performed in four consecutive iterations of 50 µL added row-wise to each well in Position 6 to prevent drying of Beads. Two rows of tips used	13.1 :Sample Plate is moved to Position 6 to allow dispensing across the entire plate	1 hour 25 min 37 sec
	13.2: Upon the addition of 50 µL agitation is applied. The 1st iteration with 30 seconds at 500 rpm. The following iterations with 250 rpm	13.2: Sample Plate is moved to Orbital Shaking Station (Position 7)	
	14: Beads are allowed to settle on the Magnetic Rack to allow subsequent supernatant removal	14: Sample Plate is moved to Orbital Shaking Station (Position 5)	1 hour 27 min
	15: Removal of supernatant in two consecutive pipetting steps using well-specific tips from tip box in Position 3 (1x 150 µL & 1x 80 µL). Dispensing in Waste Container (Position 8)	15: Supernatant is removed while remaining on Magnet (Position 5)	1 hour 28 min 40 sec
	16: Second Iteration of Steps 7.1 to 9	16: Second Iteration of Steps 7.1 to 9	1 hour 39 min 36 sec
Acetonitrile wash	17.1: Addition of 171.5 µL 100% ACN (Position 2): performed in seven consecutive iterations of 24.5 µL added row-wise to each well in Position 6 to prevent drying of Beads	17.1: Sample Plate is moved to Position 6 to allow dispensing across the entire plate	1 hour 59 min 13 sec
	17.2: Upon the addition of 24.5 µL agitation is applied. The 1st iteration with 30 seconds at 500 rpm. The following iterations with 250 rpm	17.2: Sample Plate is moved to Orbital Shaking Station (Position 7)	
	18: Beads are allowed to settle on the Magnetic Rack to allow subsequent supernatant removal	18: Sample Plate is moved to Orbital Shaking Station (Position 5)	2 hour 36 sec
	19: Removal of supernatant in two consecutive pipetting steps using well-specific tips from tip box in Position 3 (1x 150 µL & 1x 80 µL). Dispensing in Waste Container (Position 8)	19: Supernatant is removed while remaining on Magnet (Position 5)	2 hour 2 min 26 sec
On-bead Digestion	20: Addition of 35 µL 100 mM ABC (Position 2): performed in two consecutive iterations of 25 µL and 10 µL dded row-wise to each well in Position 6. Volume necessary to buffer neutral pH	20: Sample Plate is moved to Position 6 to allow dispensing across the entire plate	2 hour 6 min 50 sec
	21: Trypsin Solution (in 50 mM acetic acid) is transfered from Position 5 (Bead/Trypsin Plate) to Sample Plate (41 µL aspirated; 5 µL dispensed per well)	21: Bead/Trypsin Plate is moved to Position 5 to allow accessibility of Row B. Sample Plate is moved to Position 6 to allow dispensing across the entire plate. Subsequently Bead/Trypsin Plate is returned to Position 9	2 hour 8 min 31 sec
	22: Shaking Sample Plate for 60 seconds at 1500 rpm. In the meanwhile, the temperature block (Position 6) is heated to 37C. Leave Sample Plate for Protein Digestion at 37C.	22: Sample Plate is moved to Orbital Shaking Station (Position 5) & subsequently to Position 6 for Protein Digestion at 37C.	**2 hour 9 min 4 sec**
	23: Recover Injection-ready Peptide Samples		4 hour or ON Incubation

Appendix Protocol A. Single-step reduction and alkylation with TCEP/ CAA plus core autoSP3 clean-up.

Detailed description of main protocol tasks with step-by-step liquid handling, plate movements, and process time.

Appendix autoSP3 Protocol B: Two-step Reduction andAlkylation with e.g. DTT & CAA + core SP3 clean-up

Protocol B	Liquid Handling	Plate Movement	Total Time
Reduction & Alkylation	1: Starting Heating of Position 6 to 60°C	1: -	2 sec
	2: DTT reagent is transfered row-by-row to the Sample Plate (42 µL aspirated; 5 µL dispensed per well)	2: Bead/Trypsin Plate is moved to Position 5 to allow accessibility of Row C. Sample Plate is moved to Position 6 to allow dispensing across the entire plate.	2 min 51 sec
	3: Hanging droplet of DTT reagent is moved into the Sample Solution by shaking at 1500 rpm for 30 seconds	3: Sample Plate is moved to Orbital Shaking Station (Position 7)	3 min 22 sec
	4: 30 minutes incubation for sufficient Reduction at 60°C	4: Sample Plate is moved to Position 6	33 min 41 sec
	5: CAA reagent is transfered row-by-row to the Sample Plate (42 µL aspirated; 5 µL dispensed per well)	5: Bead/Trypsin Plate is moved to Position 5 to allow accessibility of Row D. Subsequently Bead/Trypsin Plate is returned to Position 9	35 min 37 sec
	6: Hanging droplet of DTT reagent is moved into the Sample Solution by shaking at 1500 rpm for 30 seconds	6: Sample Plate is moved to Orbital Shaking Station (Position 7)	36 min 18 sec
	7: 30 minutes incubation for sufficient Alkylation at RT	7: Sample Plate is moved to Position 6	1 hour 6 min 41 sec
Protein Binding	8: Magnetic Bead stock solution is transfered row-by-row to the Sample Plate (42 µL aspirated; 5 µL dispensed per well)	8: Sample Plate is moved to Position 6 to allow dispensing across entire plate	1 hour 8 min 44 sec
	9: Hanging droplet of Magnetic Beads is moved into the Sample Solution by shaking at 1500 rpm for 20 seconds	9: Sample Plate is moved to Orbital Shaking Station (Position 7)	1 hour 9 min 24 sec
	10: Acetonitrile (100%) is transfered from Position 2 to Sample Plate (137 µL aspirated; 15 µL dispensed per well)	10: Sample Plate is moved to Position 6 to allow dispensing across entire plate	1 hour 13 min 34 sec
	11: Proteins are allowed to bind to the Magnetic Beads while shaking in nine consecutive iterations of 30 seconds at 1500 rpm & 90 seconds at 100 rpm	11: Sample Plate is moved to Orbital Shaking Station (Position 7)	1 hour 32 min 2 sec
	12: Bead-bound Proteins are trapped on the Magnetic Rack for 5 minutes to allow subsequent supernatant removal	12: Sample Plate is moved to Magnet (Position 5)	1 hour 37 min 12 sec
	13: Removal of supernatant in two consecutive pipetting steps using well-specific tips from tip box in Position 3 (1x 18 µL & 1x 25 µL). Dispensing in Waste Container (Position 8)	13: Supernatant is removed while remaining on Magnet (Position 5)	1 hour 39 min 37 sec
Ethanol wash	14.1: Addition of 200 µL 80% Ethanol (Position 2): performed in four consecutive iterations of 50 µL added row-wise to each well in Position 6 to prevent drying of Beads. Two rows of tips used	14.1 :Sample Plate is moved to Position 6 to allow dispensing across the entire plate	1 hour 46 min 18 sec
	14.2: Upon the addition of 50 µL agitation is applied. The 1st iteration with 30 seconds at 500 rpm. The following iterations with 250 rpm	14.2: Sample Plate is moved to Orbital Shaking Station (Position 7)	
	15: Beads are allowed to settle on the Magnetic Rack to allow subsequent supernatant removal	15: Sample Plate is moved to Orbital Shaking Station (Position 5)	1 hour 47 min 41 sec
	16: Removal of supernatant in two consecutive pipetting steps using well-specific tips from tip box in Position 3 (1x 150 µL & 1x 80 µL). Dispensing in Waste Container (Position 8)	16: Supernatant is removed while remaining on Magnet (Position 5)	1 hour 49 min 21 sec
	17: Second Iteration of Steps 7.1 to 9	17: Second Iteration of Steps 7.1 to 9	2 hour 6 sec
Acetonitrile wash	18.1: Addition of 171.5 µL 100% ACN (Position 2): performed in seven consecutive iterations of 24.5 µL added row-wise to each well in Position 6 to prevent drying of Beads	18.1: Sample Plate is moved to Position 6 to allow dispensing across the entire plate	2 hour 19 min 54 sec
	18.2: Upon the addition of 24.5 µL agitation is applied. The 1st iteration with 30 seconds at 500 rpm. The following iterations with 250 rpm	18.2: Sample Plate is moved to Orbital Shaking Station (Position 7)	
	19: Beads are allowed to settle on the Magnetic Rack to allow subsequent supernatant removal	19: Sample Plate is moved to Orbital Shaking Station (Position 5)	2 hour 21 min 17sec
	20: Removal of supernatant in two consecutive pipetting steps using well-specific tips from tip box in Position 3 (1x 150 µL & 1x 80 µL). Dispensing in Waste Container (Position 8)	20: Supernatant is removed while remaining on Magnet (Position 5)	2 hour 23 min 7 sec
On-bead Digestion	21: Addition of 35 µL 100 mM ABC (Position 2): performed in two consecutive iterations of 25 µL and 10 µL dded row-wise to each well in Position 6. Volume necessary to buffer neutral pH	21: Sample Plate is moved to Position 6 to allow dispensing across the entire plate	2 hour 27 min 31 sec
	22: Trypsin Solution (in 50 mM acetic acid) is transfered from Position 5 (Bead/Trypsin Plate) to Sample Plate (41 µL aspirated; 5 µL dispensed per well)	22: Bead/Trypsin Plate is moved to Position 5 to allow accessibility of Row B. Sample Plate is moved to Position 6 to allow dispensing across the entire plate. Subsequently Bead/Trypsin Plate is returned to Position 9	2 hour 29 min 12 sec
	23: Shaking Sample Plate for 60 seconds at 1500 rpm. In the meanwhile, the temperature block (Position 6) is heated to 37C. Leave Sample Plate for Protein Digestion at 37C.	23: Sample Plate is moved to Orbital Shaking Station (Position 5) & subsequently to Position 6 for Protein Digestion at 37C.	**2 hour 29 min 45 sec**
	24: Recover Injection-ready Peptide Samples		4 hour or ON Incubation

Appendix Protocol B. Two-step reduction and alkylation with e.g. DTT/ CAA plus core autoSP3 clean-up.

Detailed description of main protocol tasks with step-by-step liquid handling, plate movements, and process time.

Appendix autoSP3 Protocol C: core SP3 clean-up (omitting automated Reduction and Alkylation)

Protocol C	Liquid Handling	Plate Movement	Total Time
**Protein Binding**	1: Magnetic Bead stock solution is transfered row-by-row to the Sample Plate (42 µL aspirated; 5 µL dispensed per well)	1: Sample Plate is moved to Position 6 to allow dispensing across entire plate	2 min 3 sec
	2: Hanging droplet of Magnetic Beads is moved into the Sample Solution by shaking at 1500 rpm for 20 seconds	2: Sample Plate is moved to Orbital Shaking Station (Position 7)	2 min 43 sec
	3: Acetonitrile (100%) is transfered from Position 2 to Sample Plate (137 µL aspirated; 15 µL dispensed per well)	3: Sample Plate is moved to Position 6 to allow dispensing across entire plate	6 min 53 sec
	4: Proteins are allowed to bind to the Magnetic Beads while shaking in nine consecutive iterations of 30 seconds at 1500 rpm & 90 seconds at 100 rpm	4: Sample Plate is moved to Orbital Shaking Station (Position 7)	25 min 21 sec
	5: Bead-bound Proteins are trapped on the Magnetic Rack for 5 minutes to allow subsequent supernatant removal	5: Sample Plate is moved to Magnet (Position 5)	30 min 31 sec
	6: Removal of supernatant in two consecutive pipetting steps using well-specific tips from tip box in Position 3 (1x 18 µL & 1x 25 µL). Dispensing in Waste Container (Position 8)	6: Supernatant is removed while remaining on Magnet (Position 5)	32 min 56 sec
**Ethanol wash**	7.1: Addition of 200 µL 80% Ethanol (Position 2): performed in four consecutive iterations of 50 µL added row-wise to each well in Position 6 to prevent drying of Beads. Two rows of tips used	7.1 :Sample Plate is moved to Position 6 to allow dispensing across the entire plate	39 min 37 sec
	7.2: Upon the addition of 50 µL agitation is applied. The 1st iteration with 30 seconds at 500 rpm. The following iterations with 250 rpm	7.2: Sample Plate is moved to Orbital Shaking Station (Position 7)	
	8: Beads are allowed to settle on the Magnetic Rack to allow subsequent supernatant removal	8: Sample Plate is moved to Orbital Shaking Station (Position 5)	41 min
	9: Removal of supernatant in two consecutive pipetting steps using well-specific tips from tip box in Position 3 (1x 150 µL & 1x 80 µL). Dispensing in Waste Container (Position 8)	9: Supernatant is removed while remaining on Magnet (Position 5)	42 min 40 sec
	10: Second Iteration of Steps 7.1 to 9	10: Second Iteration of Steps 7.1 to 9	53 min 36 sec
**Acetonitrile wash**	11.1: Addition of 171.5 µL 100% ACN (Position 2): performed in seven consecutive iterations of 24.5 µL added row-wise to each well in Position 6 to prevent drying of Beads	11.1: Sample Plate is moved to Position 6 to allow dispensing across the entire plate	1 hour 13 min 13 sec
	11.2: Upon the addition of 24.5 µL agitation is applied. The 1st iteration with 30 seconds at 500 rpm. The following iterations with 250 rpm	11.2: Sample Plate is moved to Orbital Shaking Station (Position 7)	
	12: Beads are allowed to settle on the Magnetic Rack to allow subsequent supernatant removal	12: Sample Plate is moved to Orbital Shaking Station (Position 5)	1 hour 14 min 36 sec
	13: Removal of supernatant in two consecutive pipetting steps using well-specific tips from tip box in Position 3 (1x 150 µL & 1x 80 µL). Dispensing in Waste Container (Position 8)	13: Supernatant is removed while remaining on Magnet (Position 5)	1 hour 16 min 26 sec
**On-bead Digestion**	14: Addition of 35 µL 100 mM ABC (Position 2): performed in two consecutive iterations of 25 µL and 10 µL dded row-wise to each well in Position 6. Volume necessary to buffer neutral pH	14: Sample Plate is moved to Position 6 to allow dispensing across the entire plate	1 hour 20 min 50 sec
	15: Trypsin Solution (in 50 mM acetic acid) is transfered from Position 5 (Bead/Trypsin Plate) to Sample Plate (41 µL aspirated; 5 µL dispensed per well)	15: Bead/Trypsin Plate is moved to Position 5 to allow accessibility of Row B. Sample Plate is moved to Position 6 to allow dispensing across the entire plate. Subsequently Bead/Trypsin Plate is returned to Position 9	1 hour 22 min 31 sec
	16: Shaking Sample Plate for 60 seconds at 1500 rpm. In the meanwhile, the temperature block (Position 6) is heated to 37C. Leave Sample Plate for Protein Digestion at 37C.	16: Sample Plate is moved to Orbital Shaking Station (Position 5) & subsequently to Position 6 for Protein Digestion at 37C.	1 hour 23 min 4 sec
	17: Recover Injection-ready Peptide Samples		4 hour or ON Incubation

Appendix Protocol C. Core autoSP3 clean-up (omitting automated reduction and alkylation). *Detailed description of main protocol tasks with step-by-step liquid handling, plate movements, and process time.*

Appendix autoSP3 Protocol D: Acidification and Recovery of Peptides to a new sample plate

Protocol D	Liquid Handling	Plate Movement	Total Time
**Acidification & Peptide Recovery**	1: 5% TFA is transfered row-by-row to the Sample Plate (42 µL aspirated; 5 µL dispensed per well)	1: Reagent Plate is moved to Position 5 to allow accessibility of Row E. Sample Plate is moved to Position 6 to allow dispensing across the entire plate. Subsequently Reagent Plate is returned to Position 9	2 min 45 sec
	2: Hanging droplet of 5%TFA is moved into the Sample Solution by shaking at 1500 rpm for 30 seconds	2: Sample Plate is moved to Orbital Shaking Station (Position 7)	3 min 26 sec
	3: 30 µL peptide-containing supernatant is transfered from Sample Plate in Position 5 to a new 96-well Sample Plate in Position 8	3: -	4 min 45 sec
	4: Residual supernatant is moved to the bottom of each well by by shaking at 1500 rpm for 30 seconds	4: Sample Plate is moved to Orbital Shaking Station in Postion 7	5 min 16 sec
	5: Wait few seconds until beads are settled at tube wall by the magnetic field	5: Sample Plate is moved to Magnetic Rack in Postion 5	
	6: 25 µL peptide-containing supernatant is transfered from Sample Plate in Position 5 to a new 96-well Sample Plate in Position 8 and combined with transfer round 1	6: -	6 min 42 sec
	7: Solution in new Sample Plate is moved to the bottom of each well by shaking at 1500 rpm for 30 seconds<br>8: Injection-ready peptides can be analyzed or stored	7: New Sample Plate is moved to Orbital Shaking Station in Postion 7	**7 min 37 sec**

Appendix Protocol D. Acidification and recovery of peptides to a new sample plate.
Detailed description of main protocol tasks with step-by-step liquid handling, plate movements, and process time.
